# Supplementary material for: Content-rich biological network constructed by mining PubMed abstracts
Source: BMC Bioinformatics. 2004 Oct 8;5:147. doi: 10.1186/1471-2105-5-147 (PMC528731; doi:10.1186/1471-2105-5-147)
Supplement: Additional File 2 — The original results of the above study (non-essential files are deleted to keep the file size under the limit set by BMC bioinformatics). [file 1471-2105-5-147-S2.bz2 › chilibotAdditionalFile2/dip10/86ID8202368E320/html/POU2F2_TBP.html]

 


 **POU2F2** and **TBP** 
  
Found 6 abstracts in PubMed, retrieved 6.  
 

 What does Google say? 
 PDF only 
| .edu only 

---

**Interactive relationship** (e.g. stimulation, inhibition, etc)

**Neutral relationship**- We show that the POU homeodomain of Oct2  [ **POU2F2** ]  and the evolutionarily conserved C terminal core domain of  **TBP**  are both required and sufficient for the interaction.  Ref: 8202368 Nucleic Acids Res, 1994
- The POU domains of the Oct1 and Oct2  [ **POU2F2** ]  transcription factors mediate specific interaction with  **TBP** .  Ref: 8202368 Nucleic Acids Res, 1994
- By using several different experimental systems we show that  **TBP**  efficiently associates with Oct1 and Oct2  [ **POU2F2** ] .  Ref: 8202368 Nucleic Acids Res, 1994
- Oct 2  [ **POU2F2** ]  cannot functionally interact with cloned TATA binding protein  [ **TBP** ]   **TBP**  but rather requires coactivators found in the TFIID  [ **TBP** ]  fraction.  Ref: 8428575 EMBO J, 1993
- The Oct1 POU domain, which is highly homologous to the Oct2  [ **POU2F2** ]  POU domain, likewise mediates interaction with  **TBP** .  Ref: 8202368 Nucleic Acids Res, 1994
- The interaction can also be observed in vivo, as  **TBP**  can be co precipitated with Oct2  [ **POU2F2** ]  from co transfected Cos1 cells and  **TBP**  co immunoprecipitates with the endogenous Oct1 from HeLa cells.  Ref: 8202368 Nucleic Acids Res, 1994
- In order of addition experiments, Oct 2  [ **POU2F2** ]  is required early, together with TFIID  [ **TBP** ] , to allow formation of a preinitiation complex.  Ref: 8428575 EMBO J, 1993

**Non-interactive relationship** (e.g. studied together, co-existance, homology, etc.)

- Co transfection of human  **TBP**  and Oct2  [ **POU2F2** ]  expression vectors into B cells resulted in a synergistic activation of an octamer motif containing promoter.  Ref: 8202368 Nucleic Acids Res, 1994
- We show that OBF 1 interacts in the absence of DNA with the POU domain of Oct 1 or Oct 2  [ **POU2F2** ]  and also with the general transcription factors  **TBP**  and TFIIB.  Ref: 8657574 Nucleic Acids Res, 1996
- Nine transcription factors were detected ATF 2, SRE ZBP, GATA 2, TFIID  [ **TBP** ] , Ets 1 Ets 2, E2F 1, Oct 2  [ **POU2F2** ] , p53, and AP 2.  Ref: 9542656 In Vitro Cell Dev Biol Anim, 1998
